# *In Silico* Prediction of Interactions between Site II on Human Serum Albumin and Profen Drugs

**DOI:** 10.1155/2013/818364

**Published:** 2013-03-06

**Authors:** Hideto Isogai, Noriaki Hirayama

**Affiliations:** Basic Medical Science and Molecular Medicine, Tokai University School of Medicine, 143 Shimokasuya, Isehara, Kanagawa 259-1193, Japan

## Abstract

Since binding of a drug molecule to human serum albumin (HSA) significantly affects the pharmacokinetics of the drug, it is highly desirable to predict the binding affinity of the drug. Profen drugs are a widely used class of nonsteroidal anti-inflammatory drugs and it has been reported that several members of the profen class specifically bind to one of the main binding sites named site II. The actual binding mode of only ibuprofen has been directly confirmed by X-ray crystallography. Therefore, it is of interest whether other profen drugs are site II binders. Docking simulations using multiple template structures of HSA from three crystal structures of complexes between drugs and HSA have demonstrated that most of the currently available profen drugs should be site II binders.

## 1. Introduction

Human serum albumin (HSA) which is the most abundant plasma protein binds vast array of chemically diverse exogenous and endogenous molecules [1]. Binding of a drug molecule to HSA results in increased solubility in plasma, decreased toxicity, and protection against oxidation of the bound molecule. Since HSA binding is one of the important factors which determine the ADME properties of the drugs, it is highly desirable to know the binding affinity of drugs in order to avoid undesirable drug-drug interactions. There are two approaches to predict protein-ligand interactions. Ligand-based approaches mostly use quantitative structure-activity relationships (QSARs) which are based on chemical structures and physicochemical properties of a series of compounds whose HSA binding affinities have been measured [2]. High-resolution crystal structures of HSA complexed with various molecules have shown that there are two main binding sites named sites I and II [3]. As site I is large and flexible multichamber, a variety of different molecules can bind to site I. On the contrary, ligands binding to site II are usually aromatic carboxylic acids with a negative charged group at one end of the molecule away from a hydrophobic center. Based on the reliable crystal structures, structure-based approaches are possible. The molecular docking methods, in particular, which have been largely improved recently can be applied to predict the interaction modes of drugs and the binding sites in atomic detail comparable to the experimental results. 

**Figure 1:**
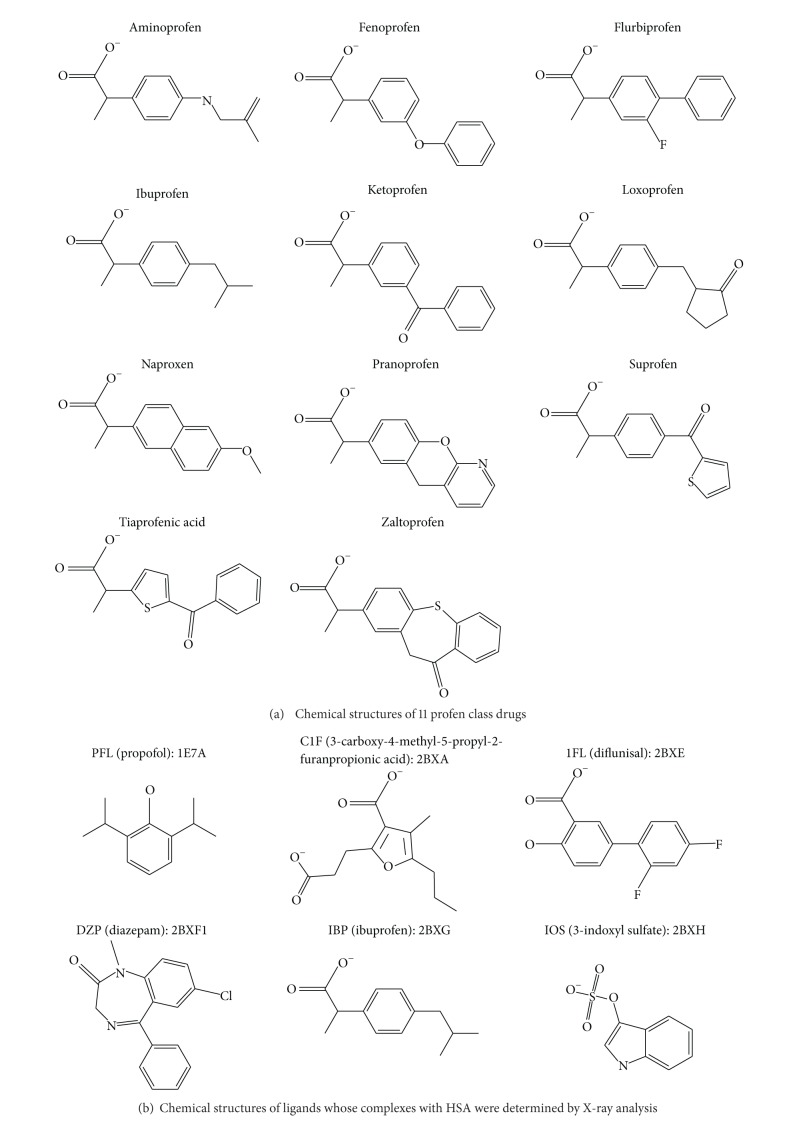

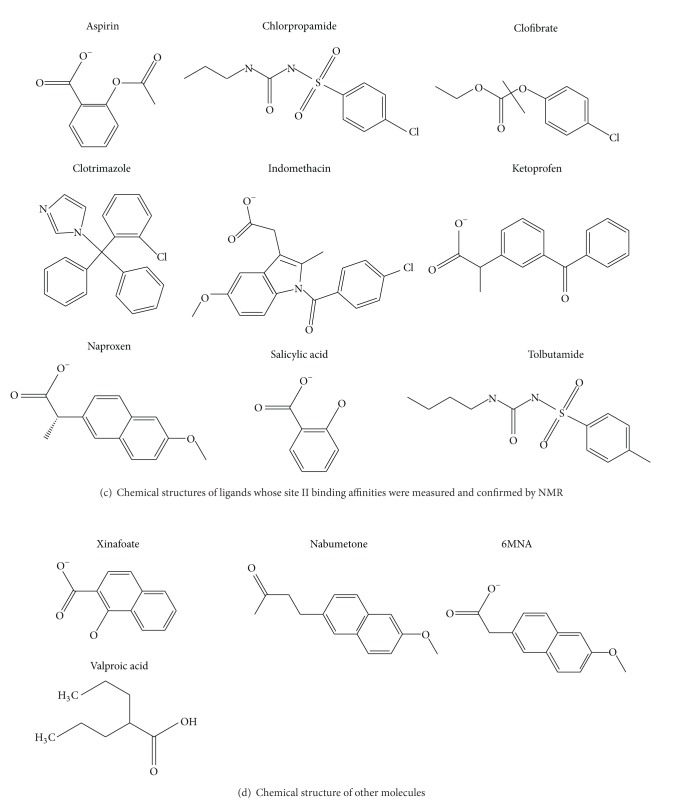
Chemical structures of drug molecules.

Since nonsteroidal anti-inflammatory drugs (NSAIDs) are among the most commonly used medications [4], the drug-drug interactions involving NSAIDs are important issues in many drug discovery projects. In particular, 2-aryl propionic acids (profen drugs) are a widely used class of NSAIDs and represent a significant share of the pharmaceutical market. In Figure 1(a), chemical structures of eleven typical profens are shown. The binding modes of flurbiprofen [5], ibuprofen [3], ketoprofen [6], naproxen [7], pranoprofen [8], and suprofen [9] have been experimentally suggested so far, albeit the exact binding mode of ibuprofen only was confirmed by X-ray crystallography. The binding site of these six profen drugs is reported to be site II. It is particularly interesting to confirm whether all of the eleven profen drugs including ibuprofen bind to site II. Therefore, in this study we have predicted whether these profen drugs can actually bind to site II by docking simulations. 

The results have shown that docking simulations can satisfactorily predict the binding of site-II-specific drugs to site II. The docking simulations undertaken in a similar way have demonstrated that all of the profen drugs shown in Figure 1(a) can bind to site II. 

## 2. Methods

### 2.1. Docking Simulations

All docking simulations were undertaken by use of software ASEDock [10]. ASEDock is based on unique concept of ASE model and ASE score. Since ASEDock is free from any bias except for shape, it is a very robust docking method. During refinement, backbone atoms of the HSA molecules were fixed and the positions of all other atoms were optimized. Docking results were evaluated by an efficiency index defined as follow:

(1) EIU
dock
=U
dock
HA,

where HA denotes the number of nonhydrogen atoms in a ligand and *U*_ dock_ = *U*_ele_ + *U*_vdw_ + *U*_strain_. Here, *U*_ele_ and *U*_vdw_ mean electrostatic and van der Waals interaction energies, respectively, between HSA and ligand molecules. *U*_strain_ refers to the difference between the conformation energy of a docked ligand and that of the minimum energy conformation nearest to the docked-ligand structure. EI_*U*_ dock_ corresponds to ligand efficiency [11]. The interaction between biotin and streptavidin is one of the strong noncovalent interactions known in nature [12]. The experimentally determined interaction energy per nonhydrogen atom is −1.6 kcal/(mol·HA) [13]. Based on this value, the threshold value of EI_*U*_ dock_ to judge site II binders was arbitrarily set to be −1.5 kcal/(mol·HA) in this study. 

A software system MOE (Molecular Operating Environment) [14] was used throughout this study. All the calculations were performed on a DELL PC workstation T7500 equipped with two Intel Xeon X5690 processors and 128 GB memory.

### 2.2. Selection of Docking Templates

Selection of suitable template crystal structures is essential for docking simulations. When we started our study, six crystal structures of complexes between HSA and different drug molecules were available. The PDB (Protein Data Bank [15]) codes are 1E7A (2.20, 0.248), 2BXA (2.35, 0.230), 2BXE (2.95, 0.226), 2BXF (2.95, 0.215), 2BXG (2.70, 0.234), and 2BXH (2.25, 0.227). The two numbers in the parentheses denote the resolution and the *R* factor, respectively. Since these six drug molecules are relatively variable in chemical structure as shown in Figure 1(b), the crystal structures reveal descent induced-fit effect around drug-binding sites. In this study, consensus-based approach was taken in order to consider the induced-fit effect by using multiple templates for docking simulations. 

Redocking simulations for these six structures have been undertaken for the validation of the algorithm of ASEDock against the HSA-drug system. Docking accuracy is usually measured by the root-mean-square deviation (rmsd) of nonhydrogen atom positions in the predicted ligand structure versus those in the crystal structure. The rmsd values (Å) obtained by redocking are 0.28, 0.70, 0.46, 0.31, 0.95, and 0.58 for 1E7A, 2BXA, 1BXE, 2BXF, 2BXG, and 2BXH, respectively. Since prediction within rmsd of 2.0 Å is held as the passing standard, the results indicate that ASEDock is suitable for the HSA-drug system.

In this study, three template crystal structures of 1E7A, 2BXA, and 1BXE which gave the smallest rmsds in the redocking simulations were selected as template structures. As the largest and the smallest drugs in size are bound to the HSA molecule in these structures, the consensus analysis using these structures as templates is expected to take the induced-fit effect into account to some extent and should be applicable to docking simulations between HSA and a variety of different molecules. 

### 2.3. Selection of Compounds for Evaluation

Since our purpose is to predict compounds which likely bind to site II, we must evaluate whether docking simulations can distinguish site II binders from other compounds. For this evaluation, we prepared three sets of compounds. The first set of 1,444 compounds mainly consists of drug molecules clinically applied in Japan now. The active metabolites of the drugs which have been reported in the literatures are also included in this dataset (DS1). Six drug molecules complexed with HSA in the crystal structures and nine additional compounds (Figure 1(c)) whose binding to site II was experimentally confirmed by NMR [16] were included in the second dataset of DS2. The compounds in DS2, 15 in total, are positive controls whose binding sites have been confirmed by X-ray crystallography or NMR. The third dataset (DS3) consists of eleven profen drugs. The binding of only ibuprofen to site II has been crystallographically confirmed. All the compounds in DS2 and DS3 are included in DS1.

## 3. Results and Discussion

**Figure 2:**
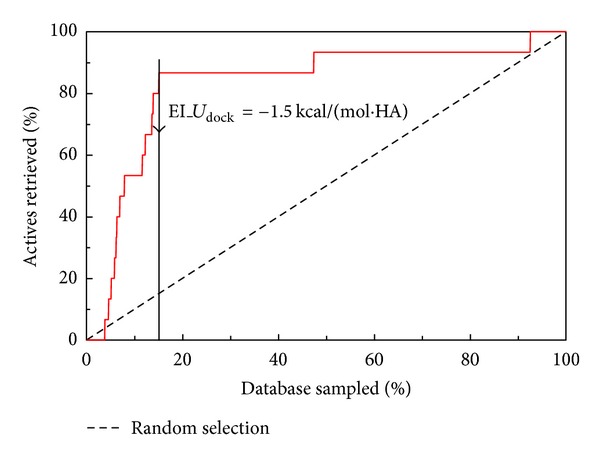
Enrichment curve for 15 site II binders. Solid line shows the predicted result.

The docking simulations between three HSA template structures and all molecules in DS1 have been undertaken. The docking results were sorted in descending order of EI_*U*_ dock _. The docked structure with the lowest EI_*U*_ dock_ value for each molecule was selected. The enrichment curve of the site II binders in DS2 is illustrated in Figure 2. The enrichment rate at the −1.5 kcal/(mol·HA) cut is 5.67. The rapid enrichment indicates that EI_*U*_ dock_ is a reasonable criterion to judge the site II binders. 13 drugs out of 15 site II binders were included in 221 compounds whose EI_*U*_ dock_ is lesser than −1.5 kcal/(mol·HA). The EI_*U*_ dock_ values for indomethacin and clotrimazole were −1.09 and −0.64 kcal/(mol·HA), respectively, and they were not judged to be site II binders by the current threshold value of EI_*U*_ dock _. Indomethacin is regarded as a site I binder in a recently published paper [17]. Clotrimazole is relatively bulkier than other 13 drug molecules and it might well be that the molecule cannot be properly accommodated at site II in the three template structures used in this study. However, from the practical point of view, the enrichment rate of 5.67 seems to be good enough to judge the site II binders. It is likely that some drugs in the remaining 208 drugs are site II binders. Therefore, the actual enrichment rate is expected to be higher than 5.67. The present study has clearly indicated that by use of the EI_*U*_ dock_ values we can distinguish site II binders in a set of compounds.

**Figure 3:**
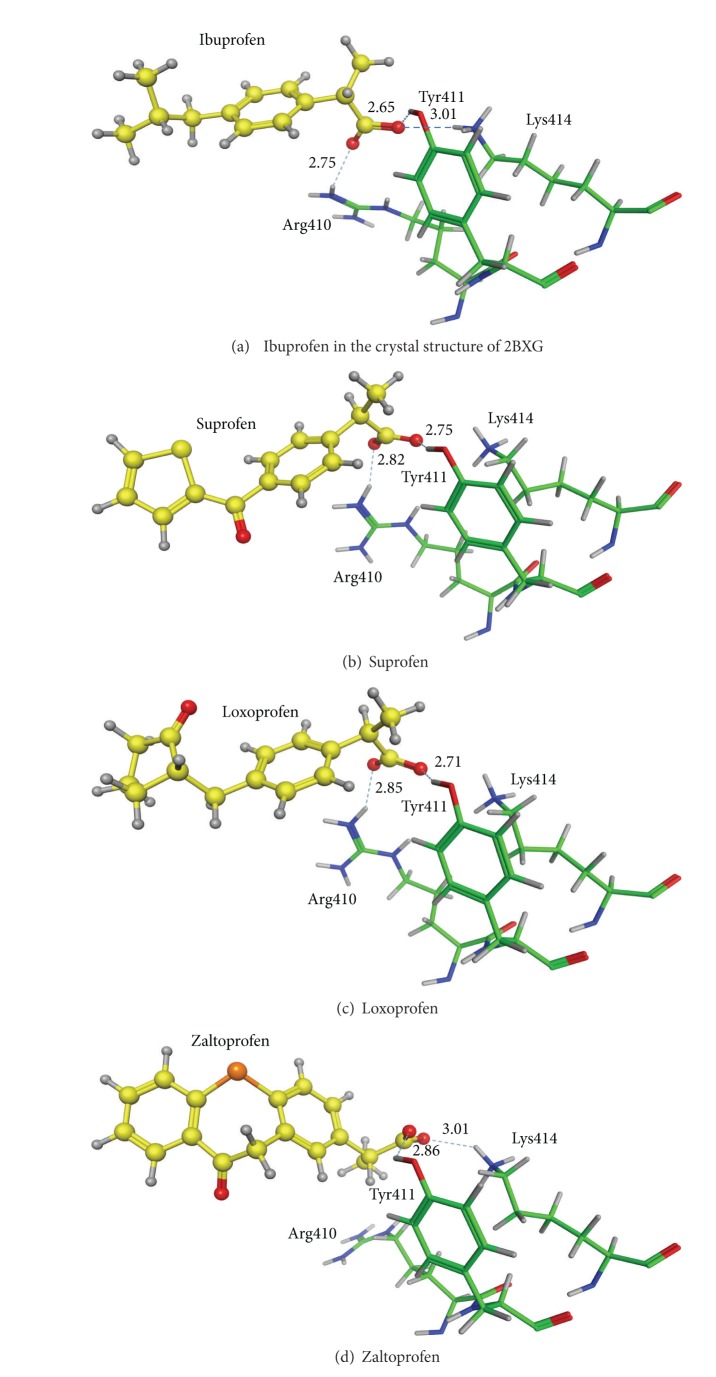
Binding modes of profens at site II. Dotted lines show hydrogen bonds with the hydrogen bond distances (Å).

The EI_*U*_ dock_ values of eleven profen drugs are given in Table 1. The EI_*U*_ dock_ values of 10 profens are less than −1.5 kcal/(mol·HA) and those drugs are considered to be strong site II binders. The exception is zaltoprofen and the EI_*U*_ dock_ value is −1.40 kcal/(mol·HA). If all these eleven profen drugs are site II binders, the enrichment rate at the −1.5 kcal/(mol·HA) cut is 5.48. The binding mode of ibuprofen at site II disclosed by X-ray analysis (PDB ID: 2BXG) is illustrated in Figure 3(a). Three amino acid residues, Arg410, Tyr411, and Lys414, specifically cluster around ibuprofen. The oxygen atoms of the carboxylic acid of ibuprofen are hydrogen-bonded to the side chains of Arg410 and Tyr411. Suprofen is reported to be a site II binder [18], but the binding mode has not been confirmed spectroscopically or crystallographically. The EI_*U*_ dock_ value of −1.67 kcal/(mol·HA) indicates that suprofen would bind to site II. The binding mode of suprofen at site II is shown in Figure 3(b). This binding mode also implies that suprofen can bind to site II. The binding site of loxoprofen has not been identified experimentally. The EI_*U*_ dock_ value of −1.53 kcal/(mol·HA) suggests that loxoprofen may bind to site II. The docking mode at site II shown in Figure 3(c) is very similar to that of ibuprofen. Therefore, loxoprofen should bind to site II. Other profens except zaltoprofen bind to site II in a similar manner. The binding affinity of zaltoprofen to site II is not comparable to those of other profen drugs, and its binding site has not been experimentally suggested so far. The binding mode of zaltoprofen is shown in Figure 3(d). The carboxyl oxygen atoms are hydrogen-bonded to Tyr 411 and Lys 414 instead of Arg410. It seems that zaltoprofen must shift marginally at site II in order to fit the bulky three-ring system to the site. As a result, Lys414 instead of Arg410 plays the role as a hydrogen-bond donor to the carboxyl oxygen atom of the drug molecule now. The binding mode clearly indicates that zaltoprofen should bind to site II. In summary, judging from the EI_*U*_ dock_ values and docking modes obtained by docking simulations, all of the profen molecules shown in Figure 1(a) are considered to be site II binders. 

**Figure 4:**
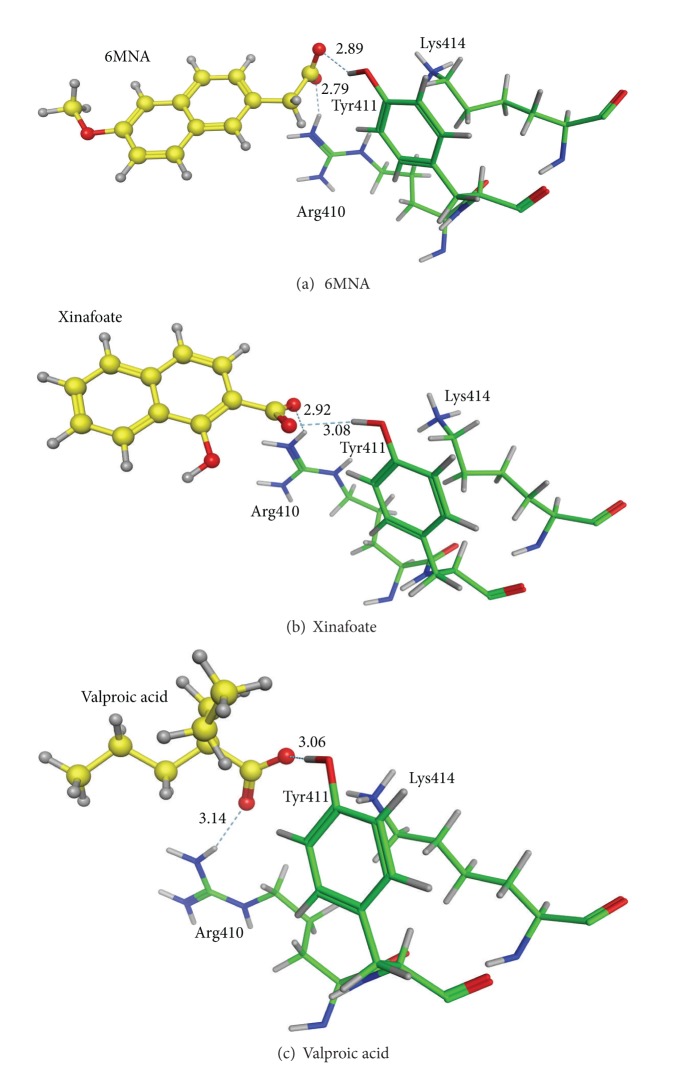
Binding modes of some other ligands of interest at site II. Dotted lines show hydrogen bonds with the hydrogen bond distances (Å).

A few noteworthy results have also been obtained. As the EI_*U*_ dock_ value of nabumetone (Figure 1(d)) is −1.45 kcal/(mol·HA), nabumetone is not judged to be a strong site II binder. However, 6MNA (Figure 1(d)) is an active metabolite of nabumetone, and its EI_*U*_ dock_ value is −1.92 kcal/(mol·HA). It suggests that not nabumetone itself but its metabolite can compete with other site II binders. In Figure 4(a), the binding mode of 6MNA at site II is shown. The specific interactions between the drug metabolite and Arg410 and Tyr411 are observed. The HSA binding affinity of xinafoate (Figure 1(d)) has not been reported so far. However, the EI_*U*_ dock_ value of −2.07 kcal/(mol·HA) and the binding mode shown in Figure 4(b) suggest the possible binding affinity of xinafoate to site II. Since xinafoate is not an active ingredient and is used as a counterion of the principal agent such as salmeterol, it might be possible that its binding affinity has been unnoticed so far. An anticonvulsant valproic acid (Figure 1(d)) which is normally used as the sodium salt was reported to be a site I binder [19]. Our work, however, has indicated that valproic acid can bind to site II, too. In Figure 4(c), the binding mode of valproic acid at site II is shown. Judging from this binding mode and the small size, it is highly expected that valproic acid binds to site II. The above results have clearly indicated that the docking simulation method employed in this study is appropriate in predicting the affinity between site II and drug molecules.

## 4. Conclusions

Since the binding of a drug to HSA is crucial for its efficacy and toxicity, evaluation of the HSA binding affinity of the drug is a particularly important issue in pharmaceutical research. The experimental measurements are time-consuming and require a lot of resources. In addition, especially in the early stage of drug discovery projects, it is highly required to synthesize compounds with appropriate binding affinity to HSA in order to avoid the future problems. In this study we have demonstrated that docking simulation can satisfactorily predict the binding of site-II-specific drugs to site II. The docking simulations have indicated that profen drugs whose binding modes have not been experimentally determined would bind to site II. Although docking simulation is a highly CPU-intensive job, the CPU time normally required is becoming not a serious problem and it can substantially substitute experimental measurements now.

## Figures and Tables

**Table 1:** The EI_*U*_ dock_ values of the intermolecular interactions between profen drugs and HAS.

Profens	EI_*U *_dock_ (kcal/(mol·HA))^1^	PDB ID
Ibuprofen	−1.92	1E7A
Naproxen	−1.89	1E7A
Aminoprofen	−1.79	1E7A
Suprofen	−1.67	1E7A
Flurbiprofen	−1.63	1E7A
Fenoprofen	−1.61	1E7A
Tiaprofenic acid	−1.59	1E7A
Pranoprofen	−1.59	1E7A
Loxoprofen	−1.53	1E7A
Ketoprofen	−1.53	1E7A
Zaltoprofen	−1.40	2BXF

^1^HA means the number of nonhydrogen atoms in a molecule.
